# Effect of nanograin–boundary networks generation on corrosion of carburized martensitic stainless steel

**DOI:** 10.1038/s41598-018-20671-z

**Published:** 2018-02-02

**Authors:** Chatdanai Boonruang, Atcharawadi Thong–on, Pinit Kidkhunthod

**Affiliations:** 1 Department of Physics and Materials Science, Faculty of Science, Chiang Mai University, Chiang Mai, 50200 Thailand; 2 Center of Excellence in Materials Science and Technology, Chiang Mai University, Chiang Mai, 50200 Thailand; 3 Synchrotron Light Research Institute (Public Organization), 111 University Avenue, Muang District, Nakhon Ratchasima, 30000 Thailand

## Abstract

Martensitic stainless steel parts used in carbonaceous atmosphere at high temperature are subject to corrosion which results in a large amount of lost energy and high repair and maintenance costs. This work therefore proposes a model for surface development and corrosion mechanism as a solution to reduce corrosion costs. The morphology, phase, and corrosion behavior of steel are investigated using GIXRD, XANES, and EIS. The results show formation of nanograin–boundary networks in the protective layer of martensitic stainless steel. This Cr_2_O_3_–Cr_7_C_3_ nanograin mixture on the FeCr_2_O_4_ layer causes ion transport which is the main reason for the corrosion reaction during carburizing of the steel. The results reveal the rate determining steps in the corrosion mechanism during carburizing of steel. These steps are the diffusion of uncharged active gases in the stagnant–gas layer over the steel surface followed by the conversion of C into C^4−^ and O into O^2−^ at the gas–oxide interface simultaneously with the migration of Cr^3+^ from the metal-oxide interface to the gas-oxide interface. It is proposed that previous research on Al_2_O_3_ coatings may be the solution to producing effective coatings that overcome the corrosion challenges discussed in this work.

## Introduction

Corrosion has had environmental and economic impacts for decades. The high cost of corrosion has been reported as approximately 3.5–4.5% of the United States’ GNP or more than 3% of the world’s GDP^1–3^. In the energy conversion and production systems of the petrochemical industry, carburizing is a long–standing corrosion problem that has been known to produce approximately 137 quadrillion joules of lost energy^3–8^. For industries that use operating temperatures of about 600 °C or higher, stainless steels are widely used. The advantage is that a protective chromia scale can form on the surface of the steel when a selective oxidation of chromium occurs under low oxygen–partial pressure. At low oxygen–partial pressure, only very stable oxides such as chromia, alumina, silica, or some spinels can form. Chromia and alumina are well known for limiting or preventing carbon transport into the alloys^5–12^. For high temperature carburizing, the carbon transport through the oxide scale can involve a diffusion of carbon–bearing molecules such as CO and CO_2_ through pores or cracks, or a diffusion of carbon through preferential diffusion pathways such as grain boundaries or even clusters of metal particles assembled as nanonetworks formed in the oxide scale. The diffusion of carbon can lead to internal carburizing followed by internal precipitation of chromium carbide, chromium depletion, and chromia–scale failure caused by brittleness, metal dusting, and even pitting corrosion^3,6,10–15^. Although martensitic stainless steel is low cost and possesses good corrosion resistance in high CO_2_ atmosphere^16^, few works have reported on carburizing–induced corrosion of martensitic stainless steel^11^ and the corrosion mechanism in nanoscale is still unclear. An understanding of the nanoscale mechanism could connect the atomic and microscale mechanisms observed during carburizing to provide a complete model of the corrosion mechanism from the initial to final states. Our current study reports on this nanoscale mechanism and the complete corrosion mechanism corresponding to surface development of martensitic stainless steel in low oxygen–partial pressured carburizing. The 2 powerful superficial X–ray techniques used to examine the surface are grazing incidence X-ray diffraction (GIXRD) and X–ray absorption near–edge structure (XANES), and the electrochemical behavior is investigated by electrochemical impedance spectroscopy (EIS). Morphology, phase, phase portion and corrosion behavior of the steel surface can be investigated using these techniques to determine the surface development and complete corrosion mechanism (atomic, nanoscale and microscale) and can lead to a solution of the corrosion problem.

The GIXRD and XANES results show the generation of a mixed Cr_2_O_3_–Cr_7_C_3_–nanograin film on top of the FeCr_2_O_4_ layer that has developed on the surface of the steel carburized at high temperature. The film possesses nanocrystallite–grain boundaries that join and form nanonetworks to transport ions involved in the corrosion reaction (i.e. Cr^3+^, O^2−^, and C^4−^). Although the increasing Cr_2_O_3_ portion and grain growth can retard bulk ion transport, the steel still encounters corrosion due to ion transport through the nanograin–boundary networks. Adding EIS results to the analysis can allow the complete carburizing–induced–corrosion mechanism to be proposed. The rate determining steps involved in the corrosion mechanism are revealed. Once the corrosion mechanism is clarified, coatings that could be effective at preventing corrosion are considered based on past research.

Tests were performed to provide information about the initial stage of carburizing. In order to save time and money, these tests were performed in a well–controlled laboratory system rather than using real long–service parts in an industrial plant environment. The carburizing of a commercial martensitic stainless steel, Fe–13.60Cr–0.33C–0.37Mn–0.28Si–0.28Ni (wt%), was done in a low oxygen–partial pressured system using the current heating technique^17^ (detailed in Methods and Supplementary Section 1). Carbon potential for carburizing is controlled by applying an electric current ranging between 100 to 300 W.

The characterization of the steel surface was performed using GIXRD, XANES, and EIS. GIXRD is a nondestructive technique providing information about crystal structure, phase composition, grain or cluster size, crystallographic interplanar spacing, lattice parameters, and residual stress in the uppermost thin crystalline layer^18–21^. XANES is powerful element specific probe of local electronic and crystallographic environment due to the absorption by inherent electron excitation and transition processes. XANES probes bonding and the local structure in a short–range order around the absorbing atoms and is particularly sensitive to the geometric arrangement of the nearest and distant neighbors of the absorbing atoms^22–25^. EIS can be used to characterize materials and indicate their conductivity and corrosion behavior. EIS works in the frequency domain and determines the net impedance in an electrochemical process through the kinetics of charge transfer and electrochemical reaction^26–29^.

## Results

**Figure 1:**
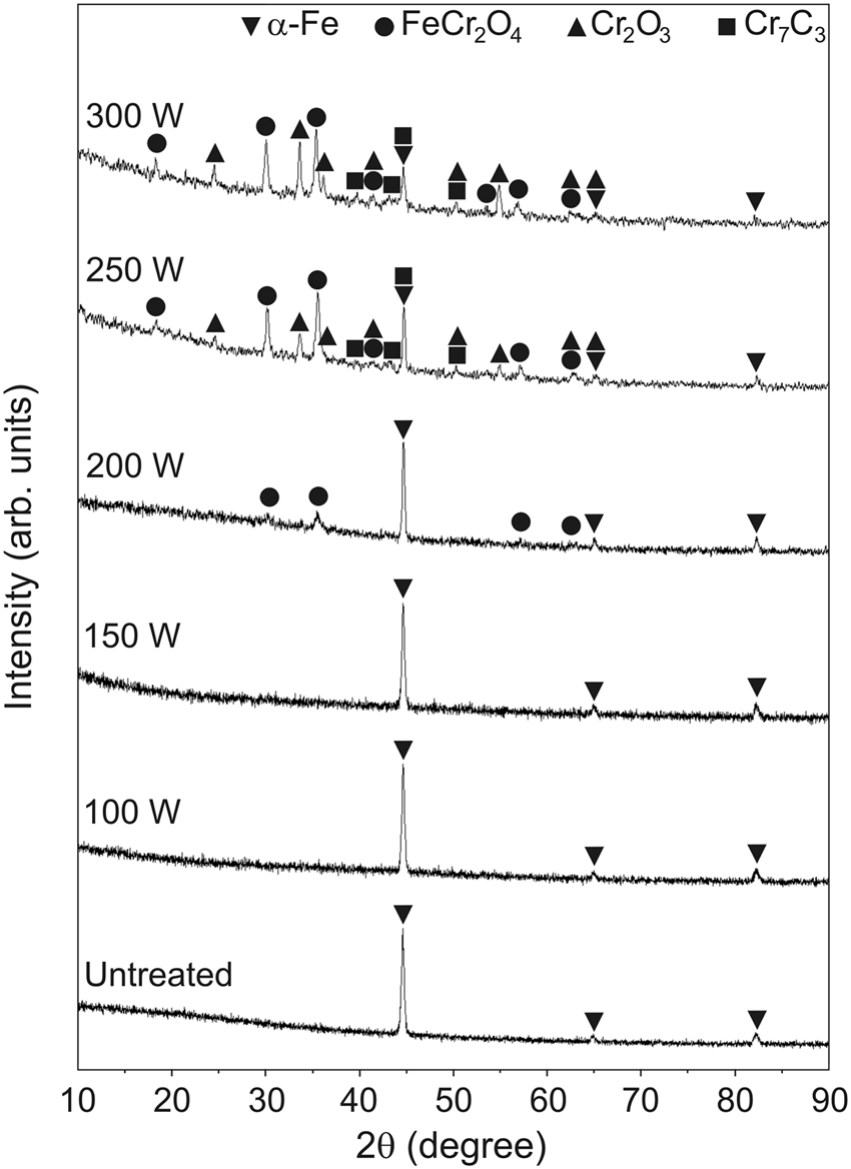
GIXRD spectra of the uncarburized and carburized martensitic stainless steels.

The high penetration depth of XRD caused high contribution of the substrate diffraction signal which diminished the signal from the surface compound therefore significant surface compound information was not obtained (Supplementary Section 2). For this reason, GIXRD was used at an average penetration depth of ~143 nm and the spectra are shown in Fig. 1. The spectra of uncarburized steel and steels carburized at 100 and 150 W show peaks solely corresponding to BCC α–Fe. For the steel carburized at 200 W, the spectrum exhibits additional peaks corresponding to FeCr_2_O_4_. The peaks of rhombohedral Cr_2_O_3_ and orthorhombic Cr_7_C_3_ were observed in the spectra of steels carburized at 250 and 300 W. Peak broadening was not observed in the spectra. The average diameters or sizes of oxide and carbide grains along [*hkl*] are in the range of 9.1 to 27.5 nm (Supplementary Section 2).

**Figure 2:**
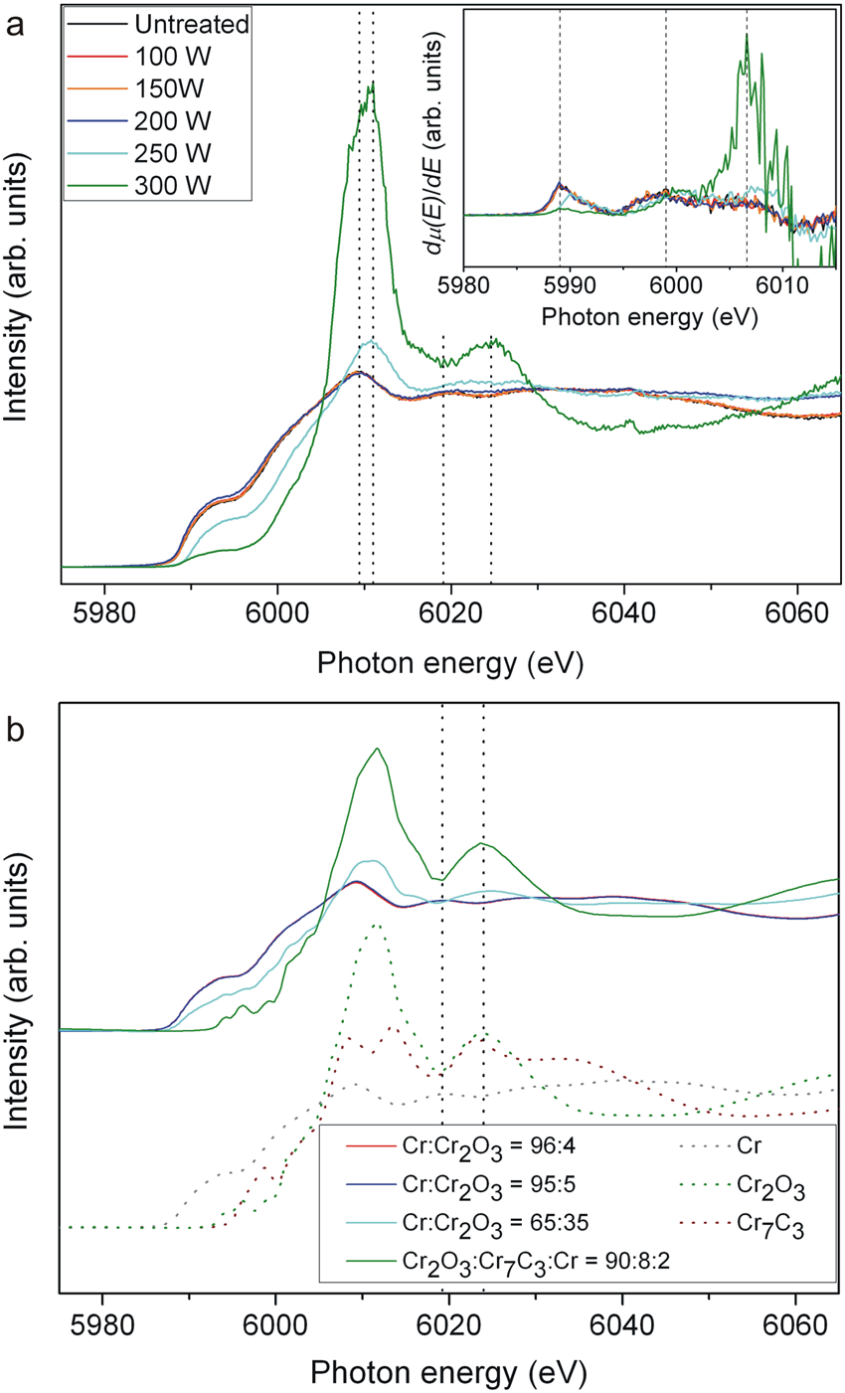
Cr K–edge Xanes experimental spectra of the uncarburized and carburized martensitic stainless steels and the associated calculated curves. (**a**) Experimental and derivative spectra; (**b**) Calculated and reference compound curves. The calculated curve for Cr:Cr_2_O_3_ ratio of 96:4 fits to both uncarburized steel and steel carburized at 100 W, while that of 95:5 fits to both steels carburized at 150 and 200 W.

In this study, Fe K–edge and Cr K–edge XANES were performed for the uncarburized and carburized steels. The Fe spectra of steels were almost identical and no additional distinguishing information was provided by the Fe K-edge results therefore only Cr K–edge spectra were reported. The experimental spectra and corresponding calculated curves are shown in Fig. 2. The curve feature analysis can be done by comparing the calculated–curve feature to those of possible forming compounds. The reference compounds include: Cr metal; Cr_2_O_3_; CrO_2_; CrO_3_; Cr_7_C_3_; and Cr_23_C_6_. Analysis determined Cr:Cr_2_O_3_ ratios of the best–fitted curves for uncarburized steel (96:4) and steels carburized at 100 W (96:4), 150 W (95:5), 200 W(95:5), and 250 W (65:35). The calculated curve with Cr_2_O_3_:Cr_7_C_3_:Cr ratio of 90:8:2 exhibited the best fit to the steel carburized at 300 W. The first and second peaks in the derivative spectra shown in the inset of Fig. 2a indicates the splitting edge energies of ∼5989 and ∼5999 eV for the steels. The spectra of steels carburized at 250 and 300 W are shown in Fig. 2a, corresponding to the curves for the Cr:Cr_2_O_3_ ratio of 65:35 and the Cr_2_O_3_:Cr_7_C_3_:Cr ratio of 90:8:2, respectively. The spectra results indicate that the steel carburized at 300 W possesses less absorption edge splitting and more white line shift which corresponds to the increased quantity of Cr_2_O_3_.

An accelerated EIS test was performed in a highly conductive electrolyte (3.5 wt% NaCl). Potential, frequency, and current were controlled and recorded using an Autolab–PGSTAT302N potentiostat (Metrohm Autolab B.V.) incorporated with NOVA software.

**Figure 3:**
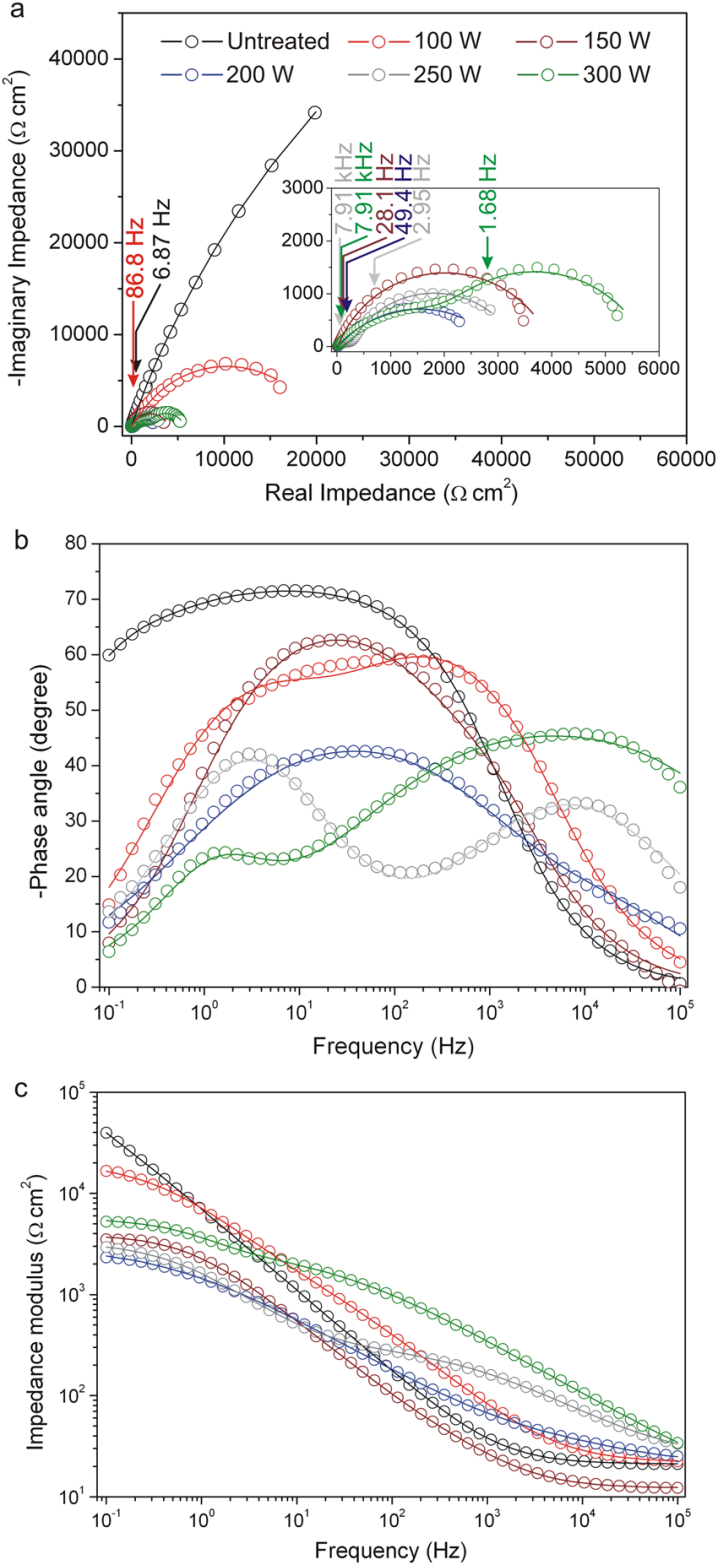
EIS experimental data (circles) and simulated curves (lines) of the uncarburized and carburized martensitic stainless steels. (**a**) Nyquist plots; (**b** and **c**) Bode plots.

The electrochemical impedance of the steel was determined by fitting an equivalent circuit to the experimental data (Supplementary Section 4). Nyquist and Bode plots in Fig. 3a–c show the simulated curves well–fitted to the experimental data. The curves of uncarburized steel and steels carburized at 100, 150, and 200 W each exhibit an arc in the Nyquist plots.

The relaxation frequencies of the carburized steels, shown in the Bode plots, were in the approximate range of 10–1,000 Hz. The relaxation frequencies of the uncarburized steel and steels carburized at 100, 150, and 200 W were 6.87, 86.8, 28.1, and 49.4 Hz, while the time constants were 23.1, 1.8, 5.7, and 3.2 ms, respectively. The curves of steels carburized at 250 and 300 W exhibited two arcs located in the low and high frequency regions in the Nyquist plots. The small arc associated with low chemical–layer capacitance (*Q*_*CPEcl*_) was observed in the high frequency region (1–10 kHz). The impedances of the steels carburized at 100, 150, 200, 250, and 300 W obtained from Nyquist and Bode plots were approximately 19, 3.8, 2.6, 3.1, and 5.5 kΩ cm^2^, respectively. Table 1 shows other parameters obtained by fitting curves using the appropriate equivalent circuit model (Supplementary Section 4).

**Table 1:** EIS parameters for simulated curves of the uncarburized and carburized steels derived from the equivalent circuit.

Steels	*OCP* (V)	*R*_*e*_ (Ω cm^2^)	CPE_cl_		*R*_*cl*_ (kΩ cm^2^)	CPE_dl_		*R*_*ct*_ (kΩ cm^2^)	*χ* ^2^
			*Q*_*CPEcl*_ (MΩ^−1^ cm^−2^ s^n^)	*n*_*cl*_		*Q*_*CPEdl*_ (MΩ^−1^ cm^−2^ s^n^)	*n*_*dl*_		
Untreated	−0.286	21.0	39.85	0.812	90.83	10.77	0.873	82.22	0.002941
100 W	−0.405	21.7	6.29	0.765	2.35	9.53	0.720	17.78	0.028943
150 W	−0.411	12.1	11.53	0.789	0.11	11.25	0.761	3.88	0.026056
200 W	−0.458	22.0	0.42	0.765	0.02	84.36	0.547	3.02	0.014924
250 W	−0.514	22.0	0.55	0.600	0.29	93.48	0.751	3.08	0.016824
300 W	−0.515	8.6	2.20	0.556	2.82	54.83	0.855	3.05	0.013679

## Discussion

Cr, O, and C atoms were dissolved in BCC α–Fe at compositions lower than the solubility limits which led the steel to remain in a single phase solid solution. For the steel carburized at 200 W, the FeCr_2_O_4_ peaks present in the spectrum indicate that the concentration of oxygen exceeded the solubility limit for steel. It is evident that the intensity of α–Fe peaks decreased and those of FeCr_2_O_4_, Cr_2_O_3_, and Cr_7_C_3_ increased with increasing applied electric power. The reaction products in this work corresponded to those of stainless steel carburized in CO–CO_2_ atmosphere which have been reported in previous works^12,13^. Since the carburizing temperature was shown to increase with increasing applied electric power (Supplementary Section 1), it is proposed that the promotion of oxide and carbide formation in the results of this work, are due to increased diffusion of oxyen and carbon. The GIXRD spectra show an unclear amorphous characteristic of peak broadening^30^ indicating a polycrystallinity of the surface compounds. The average sizes of oxide and carbide grains means the compounds can be attributed to nanocrystallites. The sizes of the compounds tended to increase with increasing applied electric power which reflects grain growth promoted by increasing temperature.

The XANES results of phase analysis were slightly different from the GIXRD results which was due to a higher surface sensitivity of XANES. There was however some correspondence found between the results from the two techniques. The XANES calculated curves for steels carburized at 250 and 300 W indicated a higher proportion of Cr_2_O_3_ than steels at a lower carburizing power which corresponded to the detection of Cr_2_O_3_ by GIXRD. The spectra for uncarburized steel and steels carburized at 100, 150, and 200 W, correspond to the calculated curves with high Cr:Cr_2_O_3_ ratios and clearly exhibit Cr metal characteristic of absorption edge splitting. On the other hand, the steels carburized at 250 and 300 W exhibit minor edge splitting. The spectra and derivative spectra show positive shifts of the white line and absorption edge (chemical shift), respectively. It is evident that the shifts increased as the portion of Cr_2_O_3_ increased. The peak energy of the Cr white line, corresponding to a 1s → 4p transition^31^, can shift from Cr metal (∼6009 eV) towards Cr_2_O_3_ (∼6012 eV) when the Cr:Cr_2_O_3_ ratios decrease. The shift contributed by the increasing average valence of the transition metal^23,31–33^ (from 0 to 3 for Cr), was promoted by the increasing amount of Cr_2_O_3_ in the steel. The development of spectral features of steels carburized at 250 and 300 W became more closely associated to those of Cr_2_O_3_ as the amount of Cr_2_O_3_ in the steel increased with increasing carburizing power. Moreover, the increase in the amount of Cr_2_O_3_ could promote the oscillation of the spectrum located at energies of ∼6019 and ∼6024 eV. This means that low oscillation features of Cr metal can develop into high oscillation features of Cr_2_O_3_. The contribution of Cr_7_C_3_ to the spectrum of steel carburized at 300 W had a significant effect on the peak energy of the white line by decreasing the contribution of the Cr_2_O_3_ white line.

This decrease resulted in a peak energy for the white line of ∼6011 eV which is 1 eV lower than that of the reference Cr_2_O_3_ at ∼6012 eV. When features of the experimental spectra are softer or smoother than that of the calculated curves, it can reflect larger amounts of nano– or amorphous constituents in the material^31^. The nano– or amorphous constituents could be revealed by XANES but not by GIXRD since amorphous characteristic features such as peak broadening were not clearly observed in the GIXRD spectra shown in Fig. 1.

The data shown in Table 1 which were based on simulated curves with *χ*^2^ ∼ 10^−2^–10^−3^, indicated that resistive and capacitive behaviors of the steels corresponded well to the Mansfeld equivalent circuit model (Supplementary Section 4). The calculated parameter of charge transfer resistance (*R*_*ct*_) was used to describe the corrosion resistance of the steel. *R*_*ct*_ of the steels tended to decrease with increasing carburizing power up to 200 W and remained constant at approximately 3 kΩ cm^2^ for the steels carburized between 200–300 W. This constant *R*_*ct*_ reflects a constant rate of redox reaction associated with the conversion process. The curves of uncarburized steel and steels carburized at 100, 150, and 200 W each exhibit an arc in the Nyquist plots. The curves reflect the occurrence of a single relaxation process in the steels. This work has shown that increasing carburizing power tended to decrease the magnitude of the arc diameter which indicates a decreasing capacitance and resistance of the steel. The relaxation frequencies of the steels suggest that they correspond to a rate determining step. It is proposed that during the EIS test, this rate determining step is that O_2_ diffusion (as uncharged electrochemically active species) in a stagnant gas layer establishes an O_2_ concentration gradient over the steel surface^34^. The time constant of steels tended to decrease with increasing carburizing power. The results exhibit the same trend as our previous work for quenched–tempered martensitic stainless steel in which the steels carburized at 100, 150, and 200 W possessed impedances of 4.4, 2.8, and 3.7 kΩ cm^2^, respectively, and slightly lower relaxation frequencies (∼10 Hz) but in the same range (10–1,000 Hz)^17^. The decreasing impedance and arc diameter correspond to a decrease in O_2_–diffusion impedance. These decreases along with a decreasing time constant are the reasons for the increasing charge transfer^35^ and decreasing corrosion resistance of the steel.

The curves of the steels carburized at 250 and 300 W each exhibit two arcs in the Nyquist plots which reflects two relaxation processes. It is proposed that these two relaxation processes were rate limiting steps in the overall process^26^. The Bode plot was used to distinguish the frequencies of the two relaxation processes and which were found to be in the ranges of 1–10 Hz and 1–10 kHz for conversion and migration processes, respectively.

The arc associated with high double–layer capacitance (*Q*_*CPEdl*_) was observed in the low frequency region (1–10 Hz) and was presumed to be associated with the accumulation of charges at the solution–oxide interface. This charge accumulation results in a decrease in the oxygen–reduction rate which corresponded to the trend noted in the gas conversion model. This model explains the generation of impedance for a redox reaction at a solution–electrode interface under the condition of uniform gas–partial pressured volume^36^. The conversion process was rate determining due to the accumulation of O^2−^ which in turn causes the oxygen–reduction rate to decrease. The conversion process was the main contributer to the charge transfer associated with *R*_*ct*_ of the steels. When the conversion process became the rate determining step at high carburizing power, *R*_*ct*_ became the important parameter determining the corrosion resistance of the steel. Outward effective diffusion of Cr^3+^ (oxidized at the steel–oxide interface) in Cr_2_O_3_ is faster than inward effective diffusion of O^2−^. The Cr^3+^ migration process is therefore rate determining for the Cr_2_O_3_ formation reaction arising at the solution–oxide interface^37,38^. The small arc, which indicates the Cr^3+^ migration through the oxide layer, has a frequency of between 1–10 kHz which corresponds to the fact that migration is dependent on the structure of the material^26^. Therefore, the migration process depends on the quantity of the compounds generated at the steel surface during carburizing and their grain sizes. Accumulation and slow migration of ions in the chemical layer can be indicated by high values of both *Q*_*CPEcl*_ and chemical–layer resistance (*R*_*cl*_). The chemical layer–relaxation frequencies (*f*_*cl*_) and calculated chemical layer–time constants (*τ*_*cl*_) for the ion migration process of the steels carburized at 250 and 300 W were approximately the same at a value of 7.91 kHz and 0.02 ms, respectively. For the conversion process, the double layer–relaxation frequency (*f*_*dl*_) and calculated double layer–time constant (*τ*_*dl*_) of the steels carburized at 250 and 300 W were approximately 2.95 and 1.68 Hz, and 54.0 and 94.7 ms, respectively. The results exhibit the same trend as our previous work for quenched–tempered martensitic stainless steel carburized at 250 W which possessed two relaxation processes with values for *f*_*cl*_ and *f*_*dl*_ of 4.5 kHz and 6.0 Hz, respectively^17^. The low *f*_*dl*_ in the low frequency arc shown in the inset of Fig. 3a indicates that the large uniform gas–partial pressured volume promoted high capacitance and a large arc diameter. The high capacitance result corresponds well to previous research^36^. It is well known that time constants and resistances have a great influence on promotion of corrosion resistance of the steel. In this work, since the *τ*_*cl*_ of the steels were about the same, therefore we propose that the *τ*_*dl*_ contributed significantly to corrosion resistance. The high–frequency arc diameter of steel carburized at 300 W shown in the Nyquist plot was larger than the diameter of the steel carburized at 250 W. This indicates higher *Q*_*CPEcl*_ and *R*_*cl*_ due to a higher quantity of Cr_2_O_3_ and larger grain sizes as previously discussed in the XANES and GIXRD sections of this work. Higher quantities of Cr_2_O_3_ and larger grain sizes can retard the migration of ions because the steel now possesses a high chemical stability and less preferential diffusion pathways for grain–boundary diffusion.

**Figure 4:**
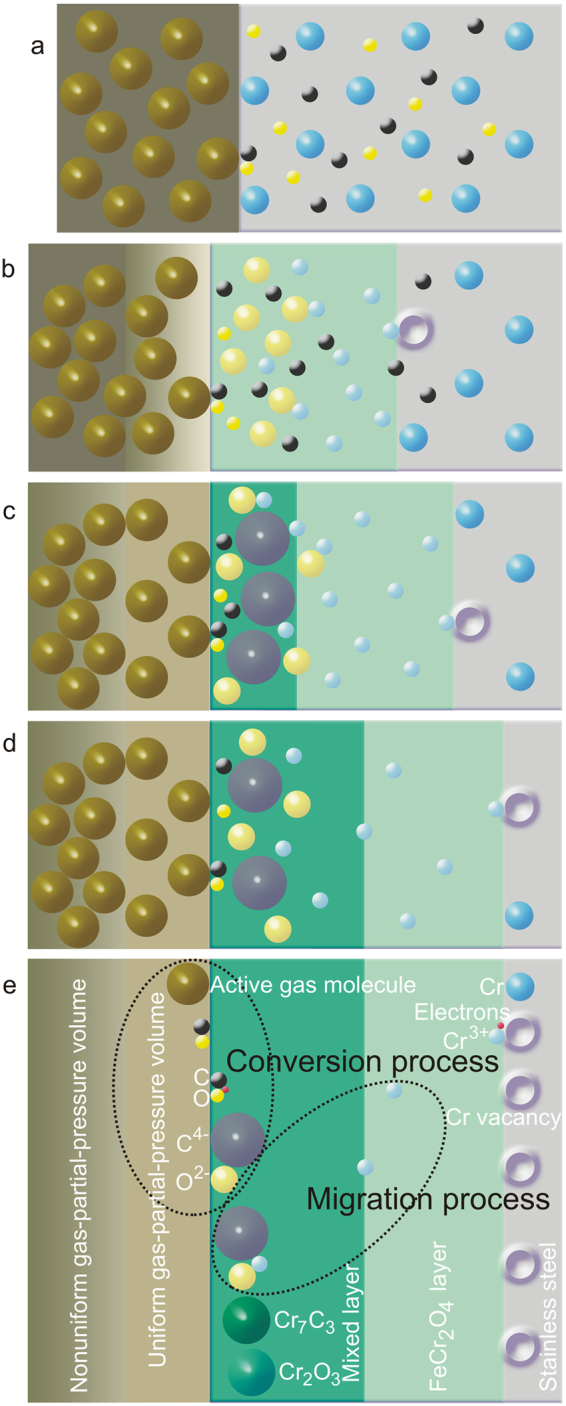
Mechanism of surface development due to carburizing martensitic stainless steel parts serviced in carbonaceous atmosphere under the conditions of low oxygen partial pressure and high temperatures. (**a**–**d**) 1^st^, 2^nd^, 3^rd^ and 4^th^ sub–stages of carburizing. The uncharged active–gas concentration in the stagnant–gas layer over the steel surface is illustrated using gradient and uniform shades to denote the corresponding gradient and uniform gas concentrations. (**e**) Schematic of the gas conversion process (adsorption and dissociation of uncharged active gas molecules/adsorption/absorption/reduction of C and O atoms) and the ion migration process. Note: A single sphere may contain one or more species. Sphere size and layer thickness in the figure do not correspond to the actual scale of the species or layer thicknesses on “in-service” parts. For simplicity, the same symbols are used for the adsorbed and the absorbed C and O atoms, and the nanograin–boundary networks are not shown.

By increasing the applied electric power for carburizing, the surface development of the steel can be compared to the stages in the carburizing process of actual “in service” parts. These parts are commonly serviced in low oxygen partial pressure in a carbonaceous atmosphere at a service temperature of approximately 600 °C or higher. The carburizing process can be divided into 4 sub–stages shown schematically in Fig. 4a–d. At the 1^st^ sub–stage of carburizing (Fig. 4a), C and O atoms dissociate from the active gas molecules adsorbing onto the steel surface. The atoms exchange electrons with the surface in a process defined as chemisorption, by breaking host–host surface bonds, creating new single bonds of carbon– or oxygen–host bonds^39^, and absorbing (or diffusing inward) into the steel through two effects: concentration gradient and thermal activation. However, single bonds are unstable consequently C and O atoms would break their bonds with host Fe atoms and then re–bond with other Fe host atoms. This effect is called bond switching which occurs during inward diffusion^39^. The overall process can be described by the following reactions^8,11–13,40–43^:

1 $$\alpha \mbox{-}{\rm{F}}{\rm{e}}+2{\rm{C}}{\rm{O}}=\alpha \mbox{-}{\rm{F}}{\rm{e}}({\rm{C}})+{{\rm{C}}{\rm{O}}}_{2}$$

2 $$\alpha \mbox{-}{\rm{F}}{\rm{e}}+{{\rm{C}}{\rm{O}}}_{2}={\rm{C}}{\rm{O}}+\alpha \mbox{-}{\rm{F}}{\rm{e}}({\rm{O}})$$

3 $$\alpha \mbox{-}{\rm{F}}{\rm{e}}+{{\rm{H}}}_{2}{\rm{O}}={{\rm{H}}}_{2}+\alpha \mbox{-}{\rm{F}}{\rm{e}}({\rm{O}})$$

4 $$\alpha \mbox{-}{\rm{F}}{\rm{e}}+{\rm{C}}{\rm{O}}+{\rm{H}}=\alpha \mbox{-}{\rm{F}}{\rm{e}}({\rm{C}})+{{\rm{H}}}_{2}{\rm{O}}$$

where α–Fe(C) and α–Fe(O) denote the ferrite matrix with dissolved carbon and oxygen, respectively. The surface activity for CO dissociation is a key factor for carbon uptake which can be reduced by H_2_O adsorption^44^, while the CO_2_ dissociation promotes oxygen uptake.

During absorption, C and O atoms occupy BCC interstitial sites of α–Fe and form unstable bonds with the host atoms, while alloying Cr atoms occupy BCC lattice sites. The compositions of C and O are less than their solubility limits leading the steel to remain in a single-phase solid solution. The redox reaction is presumed to not have occurred at this sub–stage, i.e. no electron transport from Cr to either C or O atoms which leads to a steadiness in the atomic valencies and radii of these atoms^39^. For the 2^nd^ sub–stage of carburizing (Fig. 4b), the oxygen concentration exceeds the solubility limit and so the excess oxygen atoms become thermodynamically unstable. The O atoms (with higher electronegativity than C atoms) are reduced into O^2−^ ions that react with outward diffusing Cr^3+^ ions (oxidized from the alloying Cr leaving behind atomic vacancies). The redox reaction between these ions creates stable bonds resulting in nucleation of FeCr_2_O_4_ at the steel surface. At this stage of oxide formation, the atomic vacancies can undergo self–assembly and generate nanovacancies at the oxide–steel interface^45^. The rate determining step in this reaction is suggested to be the diffusion of uncharged active gas (for example, CO_2_) in a stagnant–gas layer (shown in Fig. 4b as the gradient layer on top of the spinel layer) over the steel surface. The spinel subsequently grows and transforms into nanocrystallites with grain sizes of approximately 10 – 20 nm. The gas dissociation rate is reduced after the formation of FeCr_2_O_4_^44^. The concentration of carbon does not seem to be high enough to generate carbide at this second sub–stage. In the 3^rd^ sub–stage (Fig. 4c), FeCr_2_O_4_ nanocrystallites are formed. Nanocrystallite–grain boundaries join to form nanonetworks of transport. All diffusing species in the surface region can be transported through the preferential diffusion pathways such as these nanonetworks^11^. In this third sub-stage, the conversion process (of C into C^4−^ and O into O^2−^ at the gas–oxide interface) and the migration process (of Cr^3+^ through the oxide scale) are the rate determining steps. Cr^3+^ generated at the oxide–steel interface migrate principally through the nanograin–boundary networks and react with O^2−^ and C^4−^ to form Cr_2_O_3_ and Cr_7_C_3_ in the region near the gas–oxide interface as presented in Fig. 4e. It is suggested that Cr_2_O_3_ and Cr_7_C_3_ are nucleated at the FeCr_2_O_4_ grain boundary by these reactions^11–13^:

5 $${\rm{2Cr}}+{\rm{3O}}={{\rm{Cr}}}_{2}{{\rm{O}}}_{3}$$

6 $${\rm{7Cr}}+{\rm{3C}}={{\rm{Cr}}}_{7}{{\rm{C}}}_{3}$$

and subsequently grow to form nanocrystallites with sizes of approximately 20 –30 and 10 – 30 nm, respectively. The generation of nanograins can be promoted by the presence of water vapor in the carburizing atmosphere^44^. The mixed Cr_2_O_3_–Cr_7_C_3_ outer layer contains a higher proportion of Cr_2_O_3_ than that of Cr_7_C_3_ (as revealed by the XANES results) due to several reasons. A conversion of Cr_7_C_3_ to Cr_2_O_3_ occurs when the steel possesses high oxygen concentrations at near surface region^12,13^ and can be attributed to very low diffusion rates of carbon in Cr_2_O_3_ scale^3,5,10^, lower electronegativity of carbon than oxygen^39^, and less thermodynamic stability of Cr_7_C_3_. Due to the migration of Cr^3+^ ions, the steel can experience atomic vacancy injection at the oxide–steel interface during the growth of the mixed layer. The aggregation of injected vacancies in the form of nanocavities can lead to the formation of cavities at the oxide–steel interface promoting scale failure due to a weakening of the scale–steel adherence^45^. The migration process becomes one of the rate determining steps in the 3^rd^ and 4^th^ sub–stages (Fig. 4c and d) due to an increasing defect concentration in oxides that controls the rate of the migration^44^. In the 4^th^ sub–stage (Fig. 4d), Cr^3+^, C^4−^, and O^2−^ can still migrate through the nanograin–boundary networks in the mixed layer and the FeCr_2_O_4_ layer but at lower rates than during the 3^rd^ stage. Since the mixed Cr_2_O_3_–Cr_7_C_3_ layer forms on top of the FeCr_2_O_4_ layer in the 4^th^ sub–stage, the rates of the conversion and migration processes would be lower than in the 3^rd^ sub–stage but would still be rate determining. Carbon diffusion can lead to internal carburizing, precipitation of chromium carbide and chromium depletion resulting in chromia–scale failure due to an insufficient supply of chromium for chromia–scale formation^7,12,13^. The corrosion mechanism model can be applied not only to martensitic stainless steels but also can applicable for other high chromium ferrous alloys, i.e. high chromium steels and ferritic stainless steels.

It is evident that the steel cannot be satisfactorily protected from corrosion through the formation of a mixed Cr_2_O_3_–Cr_7_C_3_ film or even a pure Cr_2_O_3_ film. Sufficient protection can be achieved by introducing a protective film possessing much lower ion transport capabilities than a Cr_2_O_3_ film. The lower ion transport can retard the redox reaction and vacancy formation. Recent research on protective films have reported interesting findings for two coatings: Al_2_O_3_ film with metal interlayer formed by reactive magnetron sputtering; NiAl film with NiCrAlY interlayer formed by high velocity–oxy–fuel thermal spraying or low–pressure plasma spraying^4,6,15,46^. The Al_2_O_3_ coating exhibits remarkable protection since no significant evidence of any carbide or oxide was generally found under the coating. This is likely due to the fact that Al_2_O_3_ has very little or no solubility for carbon and low diffusivity for oxygen. Even though Al_2_O_3_ is more stable and resistant to carburizing than Cr_2_O_3_, carbon–bearing molecules can diffuse through pores or cracks leading to a presence of carbon in the film which hinders the protective nature of the film^4,6,15,46^. The coating of NiAl can experience generation of Ni_3_Al and Al_2_O_3_ during carburizing. The NiAl film, with Ni_3_Al and Al_2_O_3_, exhibits excellent carburizing resistance since it possesses a low carbon concentration and prevents carbide formation in the substrate by forming a compacted, defect–free and slow–growing alumina scale^4,47^.

## Conclusions

Results of this work have led to a complete model of surface development and corrosion mechanism during the carburizing of martensitic stainless steel in low oxygen–partial pressured carbonaceous atmosphere. The rate determining steps of the corrosion reaction during carburizing were determined from Nyquist and Bode plots of the EIS data. These steps are the diffusion of uncharged active gases in the stagnant–gas layer over the steel surface and the conversion of C into C^4−^ and O into O^2−^ at the gas–oxide interface simultaneously with the migration of Cr^3+^ from the metal–oxide interface to the gas–oxide interface. Throughout the carburizing process, the rate determining step changes from diffusion to conversion alongside migration due to surface development and the corrosion mechanism. The nanograin–boundary networks arising during carburizing have a great influence on the corrosion of martensitic stainless steel since they give rise to preferential diffusion pathways for ion transport even though the protective Cr_2_O_3_–containing layer is generated. As reported earlier, research have used Al_2_O_3_ coatings for protection of martensitic stainless steel because this compound can produce a defect–free coating with the characteristics of high compaction and slow growth. It is therefore proposed that this previous research on Al_2_O_3_ coatings may be the solution to producing effective coatings that overcome the corrosion challenges discussed in this work.

## Methods

### Materials and preparation

The 1.7 × 1.0 × 0.2 cm specimens were prepared from commercial martensitic stainless steel: Fe–13.60Cr–0.33C–0.37Mn–0.28Si–0.28Ni (wt%). The material was received in an annealed state (800 – 850 °C and slow cooling rate after annealing) with hardness of ∼220 HB. The microstructure of steel was constituted of randomly dispersed globular carbides in a ferrite matrix which corresponded to the steel treated in the annealed condition reported in literature^48–50^. The specimens were polished using 0.3 μm alumina and carburized using a current heating technique. This low oxygen–partial pressure carburizing technique in which the specimen was enclosed in 50 μm graphite powders, compressed to form a package with a pressure of 10.3 kPa. The package was placed in contact and between two copper electrodes connected to a 100 – 300 W DC power supply. Heat was generated by the DC current for a 30–min carburizing in a low vacuum chamber (~66 kPa absolute pressure) fed with argon (50 ml min^−1^ flow rate).

### Characterization

GIXRD was performed using D8 DISCOVER (Bruker Corporation) spectrometer with a Cu Kα X–ray source (1.5406 Å, 40 kV, and 40 mA) at a 2° incident angle, 0° tilt angle, 0.02° step size, and 0.4 s step time. Cr K–edge XANES was carried out at the BL5.2: SUT–NANOTEC–SLRI XAS beam line of the Synchrotron Light Research Institute (Public Organization), Thailand. The total fluorescence yields were collected using a double crystal Ge (220) detector. The background correction and normalization of experimental spectra were done using the Athena program of IFEFFIT package^51^ and the calculated curves were determined using FEFF8.2 codes^52,53^. The FEFF8.2 codes utilize a full multiple scattering approach based on Abinitio overlapping muffin–tin potentials. The muffin–tin potentials used in the FEFF codes are self–consistent calculations with the Hedin–Lundqvist exchange–correlation function^54^. The XANES spectra of Cr were calculated in a spherical radius of 3.4 Å for Cr_7_C_3_ and 3.7 Å for Cr_2_O_3_ around the absorber Cr atom. The full multiple scattering calculations include all possible paths within a larger cluster radius of 5.0 Å for both Cr_7_C_3_ and Cr_2_O_3_. EIS measurement was performed using the Autolab–PGSTAT302N potentiostat (Metrohm Autolab B.V.) in 3.5% NaCl solution with a Ag/AgCl reference electrode and a platinum counter electrode incorporated with NOVA (1.11.0) software. The exposure surface area of the working electrodes was approximately 0.8–1.0 cm^2^. Before EIS measurements, the open circuit potentials (OCPs) of all working electrodes were monitored for 2,000 s until stable OCPs were achieved. The applied sinusoidal potential was held to 10 mV (r.m.s.) around the OCP and the frequency was controlled to between 100 kHz and 0.1 Hz. For each analysis condition, the EIS measurement was performed for at least three times by using a new sample in fresh solution. The closest single results to the averages of multiple data were chosen to be the representation of each analysis condition. The impedance and electrochemical parameters of the steel were determined by fitting the experimental data to the Mansfeld equivalent circuit model using NOVA (1.11.0) software.

### Data availability

The datasets generated during and/or analysed during the current study are available from the corresponding author on reasonable request.

## Electronic supplementary material

Supplementary information
