# Supplementary material for: Effect of nanograin–boundary networks generation on corrosion of carburized martensitic stainless steel
Source: Sci Rep. 2018 Feb 2;8:2289. doi: 10.1038/s41598-018-20671-z (PMC5797200; doi:10.1038/s41598-018-20671-z)
Supplement: Supplementary file 1 — Supplementary information [file 41598_2018_20671_MOESM1_ESM.pdf]

Supplementary Information

Effect of nanograin–boundary networks generation on corrosion of carburized martensitic stainless steel

Chatdanai Boonruang, Atcharawadi Thong–on & Pinit Kidkhunthod

Supplementary Section 1

Energy conversion and production systems in petrochemical industry such as ethylene furnaces and hydrogen reformer plants generally encounter high temperature carburizing in carbonaceous atmosphere which results in corrosion problem[3–8,40,55]. The surface development of service parts due to carburizing is largely affected by material composition, gas content, and temperature. Types of chemical reactions at the surface, and carbon diffusion into the material are influenced by the former two parameters. The retardation of surface reaction and carbon transport through chromia scale are controlled by diffusion of chromium in stainless steel and oxide scale formation[5]. Therefore, a protection of stainless steels is greatly influenced by morphology, composition, and phase of the oxide scale. For carburizing in this study, instead of performing plant test by taking samples from real long term–used parts, a laboratory test in well–controlled system using current heating technique has been performed for the advantage of saving time and money. The current heating technique is the thermochemical–carburizing technique modified from a nanofiber–fabrication technique and a conventional–pack carburizing. In carburizing, the DC power supply is applied to the material enclosed in compressed graphite powders which results in a change of electrical energy into thermal energy, and increase in temperature and carbon–diffusion coefficient. The nascent carbon from graphite can diffuse into the material leading to generation of solid solution and carbide in the surface region of material[17,56–58]. The carburizing system is schematically presented in Supplementary Fig. 1. In this system, the carburizing was

performed subsequently by evacuation of the chamber to the absolute pressure of approximately 66 kPa, feeding Ar with the rate of 50 ml min^{-1}, and applying DC current with constant electric power to a package of material and graphite which is placed in contact between two copper electrodes for 30 min. The remaining air in chamber contributed to generate a low oxygen–partial pressured atmosphere. The relative temperatures of steels carburized at 100 – 300 W were measured by a type–K thermocouple and plotted as a function of carburizing time as shown in Supplementary Fig. 2. The temperatures of cool–down period (after 30 min carburizing) are also presented. It shows that the temperature of steels increased correspondingly to the applied electric power. This means that the high power can generate high heat–up rate and high temperature. Temperature has a great effect on a diffusion of diffusing specie in material. The diffusion coefficient (D (m^2 s^{-1})) increases exponentially with the increasing temperature as described by Arrhenius relation:

$$D = D_0 \exp\left(\frac{-Q}{RT}\right) \tag{S1}$$

where D_0 (m^2 s^{-1}), Q (J), R (J mol^{-1} K^{-1}), and T (K) are pre–exponential term, activation energy, universal gas constant, and absolute temperature, respectively. As concentration gradient of diffusing specie ($(\frac{\partial c}{\partial x})$ (m^{-4})) is approximately equal for the same system, the flux of diffusing specie through surface (J (m^{-2} s^{-1})) depends on the diffusion coefficient as described by Fick's first law:

$$J = -D(\frac{\partial c}{\partial x}) \tag{S2}$$

Therefore, the increase in temperature can promote diffusion of carbon, oxygen and chromium in the steels[56].

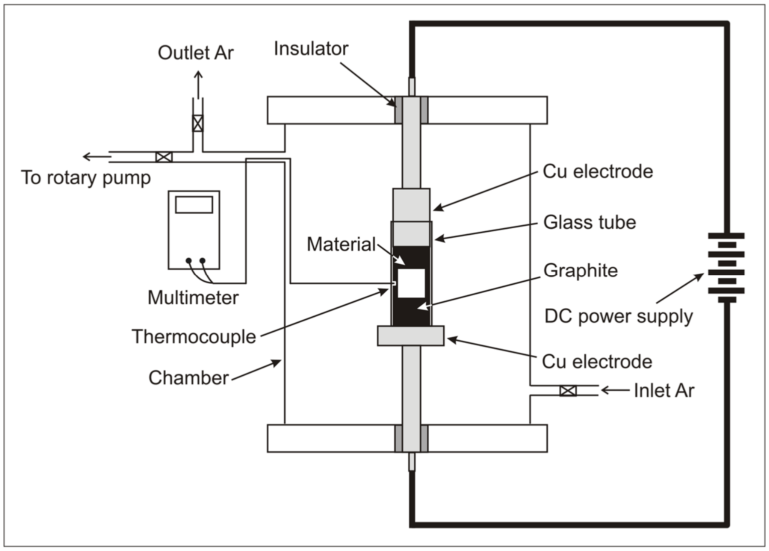

Supplementary Figure 1 | Schematic of the current heating carburizing system[17,56–58].

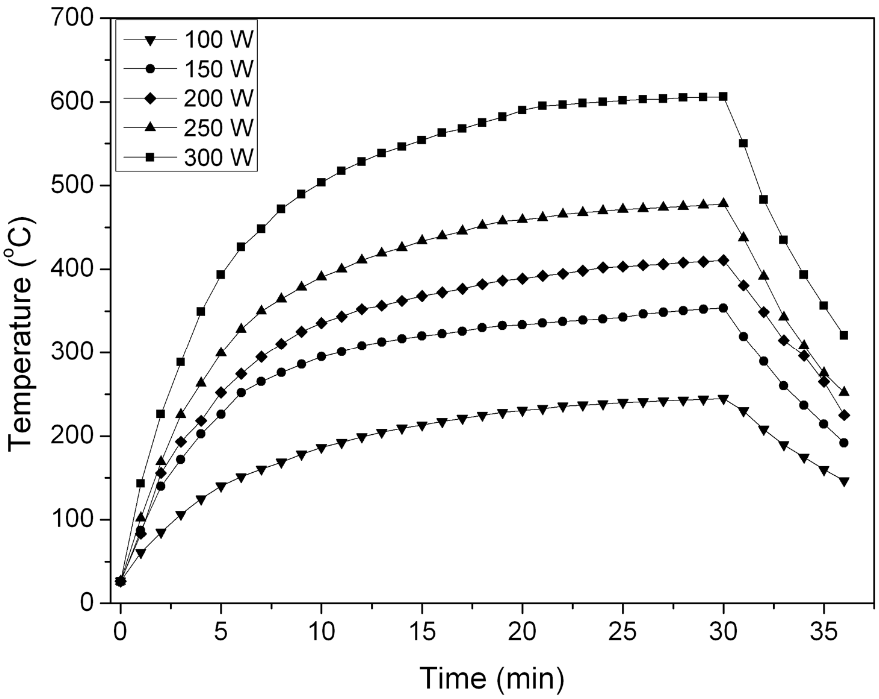

Supplementary Figure 2 | Carburizing temperature of the martensitic stainless steels as a function of time.

Supplementary Section 2

Supplementary Fig. 3 shows XRD spectra of the uncarburized and carburized steels. The spectra clearly exhibit the peaks with high substrate contribution corresponding to (110) (44.674°) and (200) (65.023°) of BCC α–Fe due to high penetration depth of X–ray. The result shows that a diffraction signal contributed by surface compounds ($FeCr_2O_4$, Cr_2O_3, and Cr_7C_3) was very low and was covered by the background. Only a weak primary peak of Cr_2O_3 corresponding to (104) (33.597°) is presented in the spectrum of steel carburized at 300 W. For this reason, with low penetration depth, GIXRD was performed to characterize the surface compound. The penetration depth of X–ray (τ) depends on a grazing incident angle. By reducing the incident angle of X–ray beam, the decreasing penetration depth and increasing absorption path of the primary beam within the compound layer itself can be achieved which lead to the increasing intensity of diffracted X–rays. A simultaneous decrease of the penetration depth keeps the impairing contribution of the substrate diffraction patterns to a minimum[21,59] and promotes the contribution of surface. The penetration depth can be determined using Snell's and Fresnel's laws which are applied to the incident angle as expressed by:

$$\tau = \frac{\cos\psi[\sin^2\theta - \sin^2(\theta - \Omega)]}{2\mu\sin\theta\cos(\theta - \Omega)} \qquad \text{(S3)}$$

where ψ, θ, Ω, and μ (cm^{-1}) are tilt angle, half of diffraction or Bragg angle, X–ray beam incident angle, and linear absorption coefficient of X–ray–irradiated material, respectively. The absorption coefficient of alloy or composite is estimated following standard practice from the weighted average evaluated from the material–elemental composition. The use of a low Ω allows the penetration depth to remain small (near the surface) even for $\psi = 0$. Each Ω yields a particular value of the mean penetration depth which is defined as the depth from which the diffracted intensity has a value of $1/e$ of the incident intensity[20,60–62]. For example, in austenitic stainless steel, the penetration depths of 26, 81, 127, and 262 nm were obtained

from the incident angles of 0.5°, 1.0°, 1.5°, and 3°, respectively[63,64]. For thin film, no crystallinity detection can be occurred when film thickness is smaller than the penetration depth[65]. The diffraction pattern can provide the information of peak position and relative peak intensity to identify crystalline phases with different orientations. A sharp and strong peak reflects enhanced film crystallinity. Moreover, a peak broadening or no detectable peak can be an indication of a presence of amorphous or nanocrystalline phase[19,30,66–68]. The peak broadening is mainly due to co–presence of different phases with random orientations which can make the diffraction pattern complicated[59]. However, the pattern cannot provide information about bonding and local structure of amorphous and nanocrystallite.

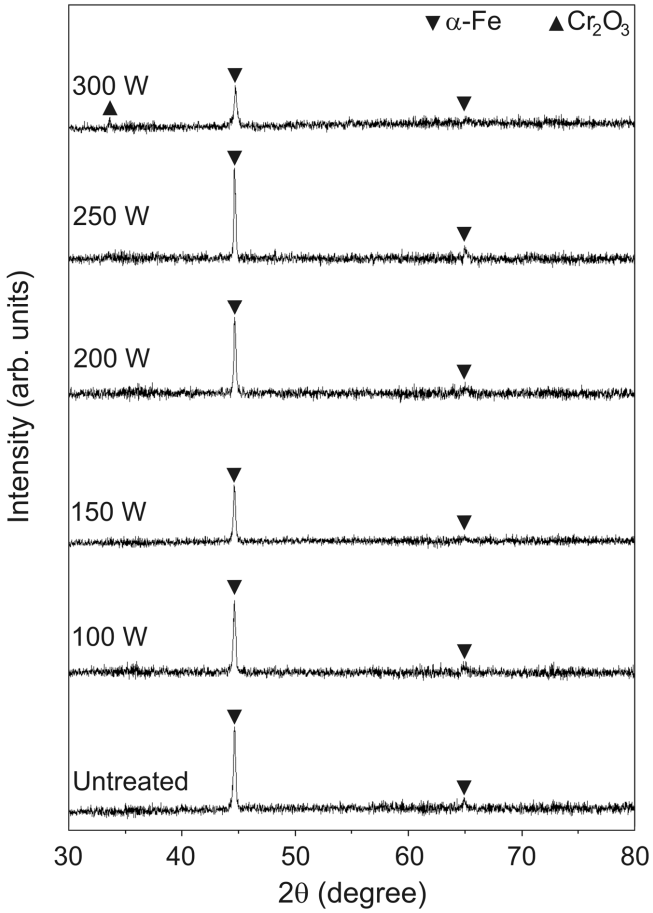

Supplementary Figure 3 | XRD spectra of the uncarburized and carburized martensitic stainless steels.

In this study, GIXRD promoted a diffraction signal from the penetration depth of ~143 nm by using the monochromatic X–ray with the wave length, incident angle, and tilt angle of 1.5406 Å, 2°, and 0°, respectively. The promotion of signal resulted in a detection of the surface compounds as shown by the spectra in Fig. 1. The spectra of uncarburized steel and steels carburized at 100 and 150 W show the peaks solely corresponding to (110) (44.674°), (200) (65.023°), and (211) (82.335°) of BCC α–Fe. For the steel carburized at 200 W, the spectrum exhibits additional peaks corresponding to (220) (30.147°), (311) (35.510°), (400) (43.161°), (511) (57.071°), and (440) (62.671°) of $FeCr_2O_4$. The additional peak of (111) (18.319°) of the spinel is present in the spectra of steels carburized at 250 and 300 W. The presence of a peak corresponding to (111) reflects stronger signal coming from a large interplanar spacing (d_{111}) of these steels in comparison with the steel carburized at 200 W. The peaks of rhombohedral Cr_2O_3 corresponding to: (012) (24.494°); (104) (33.597°); (110) (36.196°); (113) (41.480°); (024) (50.220°); (116) (54.852°); (214) (63.449°); and (300) (65.106°), and orthorhombic Cr_7C_3 corresponding to: (150) (39.673°); (112) (43.038°); (151) (44.833°); and (222) (50.978°) were observed in the spectra of steels carburized at 250 and 300 W. The results show that even though a primary peak of Cr_7C_3 (151) (44.833°) is superimposed by the α–Fe (110), the next three minor peaks can indicate the formation of Cr_7C_3. A presence of carbon–rich Cr_7C_3 carbide without $Cr_{23}C_6$ carbide indicated that the steel surface had high carbon concentration and high cooling rate after finished the carburizing. The high cooling rate can suppress diffusion of Cr and C, and transformation of Cr_7C_3 carbide into the stable $Cr_{23}C_6$ carbide[69]. The high oxygen concentration for the steel carburized at 250 and 300 W could reduce the diffraction signal from corresponding small d_{hkl} planes, e.g. of $FeCr_2O_4$, due to the change of d_{hkl} or local lattice disorder[19] caused by occupying oxygen located at between the planes. Meanwhile, the large d_{111} (4.839 Å) of $FeCr_2O_4$ underwent smaller change and remained exhibited strong signal. The diffraction

signal corresponding to a large d_{150} (2.297 Å) of Cr_7C_3 for the steel carburized at 300 W can be described in the same way. On contrary to the carburizing of austenitic stainless steel in literature[3], no X–ray–peak shift of α–Fe was observed which indicated that no metal–particle formed in the steel in this study. It is proposed that this resulted from the different material and carbonaceous–medium composition which determined type of chemical reaction and quantity of carbon diffusion.

For a penetration depth calculation using equation (S3), a half of diffraction angle and linear absorption coefficient of the stainless steel were determined from the strongest non–overlapping peak of compound in the spectrum and the steel composition, respectively. In this study, the depth of ~143 nm was determined from the average value of the depths calculated from $FeCr_2O_4$, Cr_2O_3, and Cr_7C_3 peaks. For composite, an intensity of diffraction pattern results from a contribution of various phases. Quantitative information about the types of involving phases and their portions can be obtained from the pattern by utilizing a deconvolution technique assuming Gaussian shapes of the constituent peaks from various phases[59]. For surface–diffusion modified alloys, the intensity is influenced by oxidation–rate constant, diffusion coefficient of diffusing specie, and diffusion time[70]. However, a presence of nonstoichiometric compound (caused by a generation of a large number of vacancy) and local lattice disorders (occurred during the modification) may lead to a peak broadening and an obvious reduction in intensity or even disappearance of the XRD peaks corresponding to some lattice planes[19,71]. For low strain material, cluster (or grain) size and interplanar spacing (or interatomic–layer distance) of the compound cluster can be evaluated using Scherrer's and Bragg's equations, respectively, as follows:

$$D_{hkl} = K\lambda/\beta_{hkl}\cos\theta \quad \text{(S4)}$$

$$d_{hkl} = \lambda/2\sin\theta \quad \text{(S5)}$$

where D_{hkl} (Å), K, λ (Å), β_{hkl} (rad), θ (degree), and d_{hkl} (Å) are average diameter of compound cluster (or grain) along the [hkl], Scherrer's constant (0.9 for assuming to be a spherical shape), wavelength of X–ray, full–width at half–maximum of the (hkl) diffraction, half of diffraction angle, and interplanar crystal spacing (or d–spacing) of the (hkl), respectively. Lattice parameters of the compound can be determined from the interplanar spacing and coordination geometry[18,19,72,73]. Equation (S4) shows that the broad peak (with high β) indicates the small size of crystalline domains[74]. Average diameters (or sizes) of the compound clusters (or grains) along the [hkl] (calculated using equation (S4)) for the steels are presented in Supplementary Table 1. Some parameters used for calculation (β_{hkl} and θ) were obtained from the strongest non–overlapping peak of compound in the spectrum. For example, β_{104} (7.19 mrad) and corresponding θ (16.81°) of Cr_2O_3 were obtained from the peak corresponding to (104) as presented in Supplementary Fig. 4. The grain size of $FeCr_2O_4$ seemed to be not increase after generation of Cr_2O_3 grains, while the grains of Cr_2O_3 and Cr_7C_3 tended to grow as the applied electric power increased. It is believed that a generation of Cr_2O_3 at $FeCr_2O_4$ grain boundaries can suppress the growth of $FeCr_2O_4$ grains by the transformation of $FeCr_2O_4$ to Cr_2O_3.

Supplementary Table 1 | Average diameters of $FeCr_2O_4$, Cr_2O_3, and Cr_7C_3 grains along the [*hkl*] for the steels carburized at 200 – 300 W calculated from GIXRD spectra

Carburizing Condition (W)	D_{311} of $FeCr_2O_4$ (nm)	D_{104} of Cr_2O_3 (nm)	D_{112} of Cr_7C_3 (nm)
200	14.6	-	-
250	20.4	20.1	9.1
300	19.8	27.5	24.6

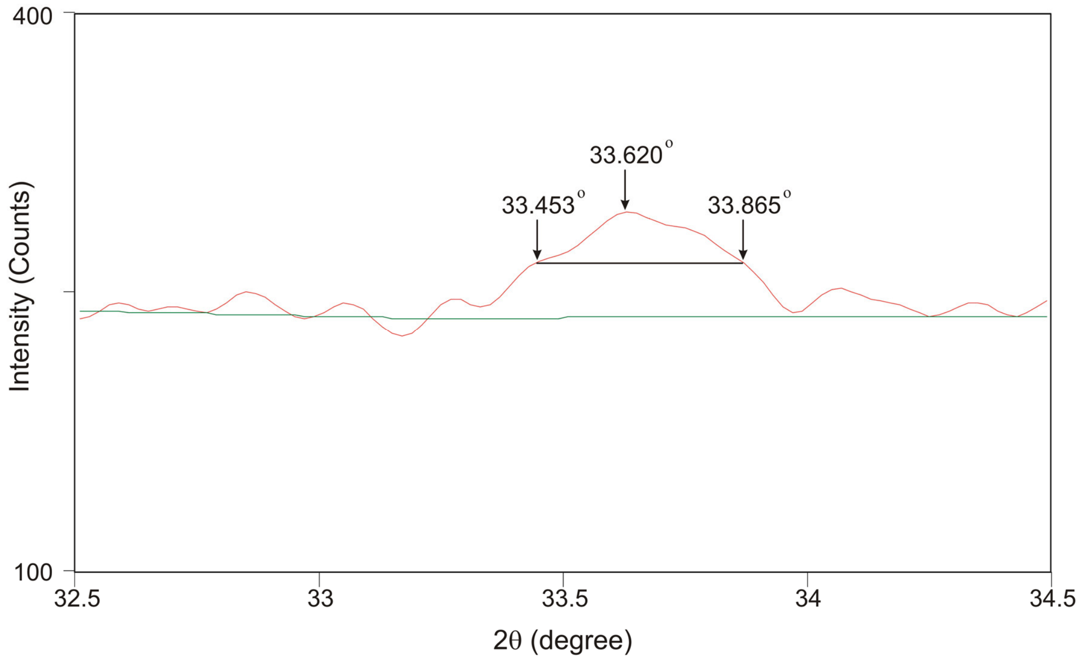

Supplementary Figure 4 | Evaluation of β_{104} and 2θ of Cr_2O_3 from the peak corresponding to (104) in GIXRD spectrum of the steel carburized at 250 W.

Supplementary Section 3

X–ray absorption near–edge structure (XANES) can provide information about distribution of the atoms of specific element between two phases. XANES represents a spectrum which is used to simulate a sum of contributions from all absorbing atoms of specific element. The simulation has been applied to not just the atoms in well–ordered crystallites but also in complex materials such as nanocrystallites or amorphous. The simulation can be made as a linear combination of the two phases which lead to obtain a proportion of the specific atoms in each phase[25,31,32,75]. XANES results from the absorption of X–ray photons generated by strongly localized electronic excitations of core electrons in ground state into unoccupied excited states above the Fermi level leaving behind core holes. The excited electrons or secondary electrons generated from absorbing atom can have inelastic scattering processes, including single– and multiple–scatterings, by neighboring

atoms. The X–ray absorption can be quantitatively expressed in terms of a linear absorption coefficient ($\mu(E)$) of a monochromatic X–ray photon with an energy E, as expressed by:

$$\mu(E) = \mu_0(E)(1 + s(E) + m(E)) \qquad \text{(S6)}$$

where $\mu_0(E)$, $s(E)$, and $m(E)$ are atomic absorption, signal due to single–, and multiple–scatterings, respectively[23,24,76–78]. The absorption of X–ray in matter increases abruptly when photon energy reaches the ionization potential of inner–shell electron. This increase can lead to the generation of strongest intensity feature in XANES spectrum which is called white line. The intensity of white line depends on bonding interaction and average number of unoccupied states (higher intensity indicates stronger interaction and higher valence). The peak energy of white line also indicates the valence because the energy cost for creating a core hole depends on the screening of valence electrons (higher energy indicates higher valence state)[23,24,33,76]. For this reason, the feature of XANES spectrum which include: absorption edge; characteristic of main peak and pre–edge; and normalized absorption intensity, can provide a particular information about the chemical state and the geometry of coordination polyhedron of the specific ion in compounds. The information include: oxidation states of ions; local ion replacement; coordination number; charge transfer; bond length; and presence of vacancy in a first shell of coordinating atoms surrounding the absorbing atom[24,31,33,75,76,78,79]. The structural information of probing element can be extracted by comparing an absorption feature of unknown matter to those of well–known reference compounds. A proportion of each compound in the investigated material can be obtained based on a linear combination of the XANES spectra of reference compounds[22,76].

In this study, the spectrum feature can give information about local crystal structure near chromium atom/ion in any compound. The pre–edge feature of the calculated curve for Cr_2O_3:Cr_7C_3:Cr ratio of 90:8:2 represents the change of valence state contributed by a charge transfer from Cr atom to ligand (O^{2-} or C^{4-}) during bonding or 3d–2p hybridization between

Cr and O in Cr_2O_3 or Cr and C in Cr_7C_3[31]. Inset of Fig. 2a shows a chemical shift from the second absorption of Cr metal (~5999 eV) toward the absorption edge of Cr_2O_3 (~6007 eV) reflected in the spectra of untreated steel and steel carburized at 300 W, respectively. The chemical shift indicated higher ionization of the chemical state of absorbing metal atoms[31,79] when oxygen content increased. The intensity of Cr white line shown in Fig. 2a increased with increasing Cr_2O_3 portion which indicated increasing Cr valence or stronger interaction between Cr^{3+} and O^{2-} in Cr_2O_3 than between Cr atoms in Cr metal[23,80]. The calculated curve for Cr:Cr_2O_3 ratio of 65:35, shown in Fig. 2b, possesses a broad white line which can indicate a linear combination of Cr metal and Cr_2O_3 white lines. The broadening is promoted by higher proximity of Cr and Cr_2O_3 quantities in comparison with the other spectra. The curve feature of steels may be different from those of reference compounds due to a presence of oxygen vacancy in the first coordination sphere of Cr atom or a presence of some impurity in the steel.

Supplementary Section 4

EIS is a non–destructive technique which can provide time–dependent information about the electrode processes and complex interfaces by extraction some characteristics of materials such as oxide coatings or paintings which are high resistance materials. The usefulness of EIS is that it can distinguish the dielectric and electric properties for each contribution of components under investigation[81]. The frequency range of EIS may probe and span transfer processes which occur in electrochemical process. The processes include: the diffusion process or mass transfer, corresponding to the mHz–range frequency, which occurs over time scales of seconds to hours; the electron transfer, corresponding to the MHz–range frequency, which occurs over much faster time scales; and the ion transfer which occurs over time scales between the previous two processes. EIS can measure current which is contributed from diffusing species and electrons. The current is a frequency–variable response of an

electrochemical system to an applied voltage[29]. In the measurement, a constant DC potential is applied to the system to set the surface concentrations of the oxidizer and reducer. The DC potential is in addition to small–amplitude sinusoidal or AC potential of a given frequency to induce small perturbations, usually in the range of 5 – 20 mV, to the equilibrium. The current–response perturbation through the electrochemical cell is recorded[26,81]. The AC potential and resulting AC current are out of phase with each other, with a phase angle of ϕ, due to capacitance of material surface which can lead to yielding a complex impedance called faradaic impedance (Z)[29]. The Faradaic impedance is constituted of real (Z') and imaginary (Z'') components corresponding to passive (resistive) and reactive (capacitive and/or inductive) contributions, respectively. The impedance constitution and phase angle between Z' and Z'' are given by:

$$Z = Z' + Z'' \tag{S7}$$

$$\phi = \tan^{-1} \frac{Z''}{Z'} \tag{S8}$$

Electrochemical process in many systems generally possesses more than one rate determining step. Each step represents an impedance component of system and contributes to an overall reaction–rate constant. The impedance of component is fitted to an equivalent circuit which is an integrated resistor–capacitor–inductor circuit model. The fitting is done for extracting parameters (such as specific resistance, capacitance, and inductance) with an aim to understand the relevant electrical and electrochemical processes. The equivalent circuit for system possessing more than one rate determining step therefore contains more than one component[28,29,81–83]. For a simple system, charges of two different polarities attracted to and stored on an electrode surface can generate a double electric layer. The double layer possesses ohmic (or electrolyte) resistance (R_e), polarization (or charge transfer) resistance (R_{ct}), and double layer capacitance (C_{dl}) of electrochemical process which corresponds to the Randles equivalent circuit model (Supplementary Fig. 5a)[29,81]. The resistance or capacitance of each

component in the circuit is affected by electrode, electrode–electrolyte interface, electrode–redox–couple interactions, etc. and can be used to characterize electrochemical performance of material. For more corresponding to the electrochemical performance, a capacitor in the circuit is replaced by a constant phase element (CPE). The element is a non–ideal capacitor whose characteristic is deviated from ideal capacitor by the effects of surface defects, non–uniform potential and current distribution, local charge heterogeneity, roughness, composition variations, etc. For any given range of angular frequency (ω) and fitting parameter (Q), the electrical impedance as a function of the frequency ($Z(\omega)$) is described as:

$$Z(\omega) = \frac{1}{(Q\omega)^{\Theta}}[\cos(\frac{\Theta\pi}{2}) - j\sin(\frac{\Theta\pi}{2})]; \quad \text{where } \Theta = \frac{2}{\pi}\cot^{-1} - \frac{Z'}{Z''} \tag{S9}$$

The Θ values of 0, 1, and -1, correspond to resistive, capacitive, and inductive behaviors, respectively[29,81]. The impedance of CPE is therefore expressed as:

$$Z_{CPE} = \frac{1}{Q_{CPE}(j\omega)^n} \tag{S10}$$

where Q_{CPE}, j, and n are capacitance of CPE, $\sqrt{-1}$, and degree of deviation from ideal capacitor, respectively[17,81,84,85]. Value of n is generally between 0.5 and 1. A CPE with $n \geq 8$ is attributed to a capacitor[86]. For a system with transport phenomena in electrolyte, the faradaic impedance is constituted of kinetic and diffusion impedances. The diffusion impedance describes a contribution of concentration polarization (or overvoltage) which depends on transport phenomena in the solution. In the absence of convection, it is referred as Warburg impedance (Z_W) with a phase angle (ϕ) described as[81]:

$$Z_W = Z'_W + Z''_W \tag{S11}$$

$$\phi = \tan^{-1}\frac{Z''_W}{Z'_W} = -1 \tag{S12}$$

For an electrochemical system of metallic material with oxide film, a chemical capacitance (or pseudocapacitance or Faradaic capacitance) is another important parameter

widely used to measure a contribution of oxygen transfer through bulk material. It is a non–interfacial capacitance arising from a change of oxygen stoichiometry in a mixed ionic–electronic conducting oxide film. The oxygen reduction in the film can be divided into two steps: (i) reduction from O_2 to O^{2-} at the gas–film interface; and (ii) migration of oxide ions through a bulk of film. For a given situation, either of these two steps can be rate determining. In thin film electrodes, when these chemical–reaction steps are rate determining, the accumulation of electroactive intermediate species in the bulk can increase the effective capacitance. The chemical capacitance is proportional to a film thickness and can promote the change in bulk stoichiometry[16,81].

In EIS measurement, the measured impedance (Z) is therefore expressed to be the sum of electrochemical parameters: electrolyte resistance (R_e) + chemical impedance ($Z_{chemical}$) + electrochemical kinetic impedance ($Z_{interface}$). $Z_{chemical}$ corresponds to film or chemical–layer resistance (R_{cl}) and chemical–layer capacitance (Q_{CPEcl}). $Z_{chemical}$ arises from oxygen absorption and diffusion in solid and fluid phase both inside and outside the electrode. $Z_{interface}$ corresponds to charge transfer resistance (R_{ct}) and double–layer capacitance (Q_{CPEdl})[26]. For the system of metallic material with oxide film, $Z_{chemical}$ corresponds to ion–migration and uncharged–diffusion processes, and $Z_{interface}$ corresponds to a conversion process[26,34,36]. The equivalent circuit corresponding to such system is the Mansfeld model[16,81,87,88] shown in Supplementary Fig. 5b. The model is extensively used for stainless steels whether with passive film or coatings. The equivalent circuit constitutes of a parallel arrangement of a charge transfer resistor (R_{ct}), and a double layer–constant–phase element (CPE_{dl}). These two components are series–connected to a chemical layer resistor (R_{cl}). The three components are connected in parallel with a chemical layer–constant–phase element (CPE_{cl}). These four components are series–arranged with an electrolyte resistor (R_e). For stainless steel without passive film due to film has been removed or cannot be generated, the Randles model is an appropriate equivalent circuit. For examples: a martensitic stainless steel electrode which was

applied strong cathodic polarization for 0.5 h for removal of passive film before EIS measurement; or a martensitic stainless steel which was EIS–measured in deaerated chloride solution[84,89].

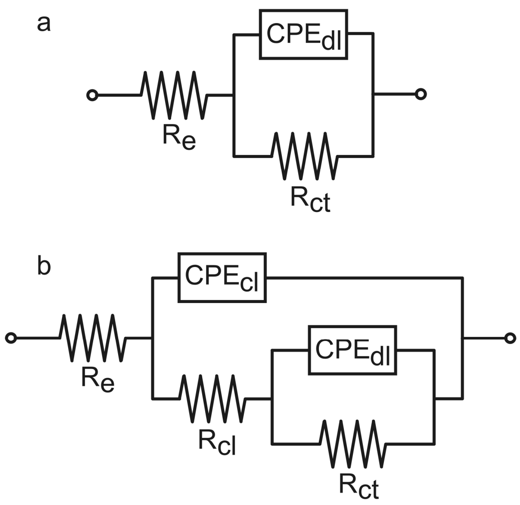

Supplementary Figure 5 | Electrochemical impedance equivalent circuits. a, Randles and **b**, Mansfeld models.

The favorite representations for EIS are Nyquist plot and Bode plot. The Nyquist plot is a plot of Z'' vs. Z' which can provide information about kinetics of electrochemical processes. Generation of semicircle or arc in The Nyquist plot indicates relaxation (or charge polarization) and charge transfer processes in the electrochemical response of material. The relaxation includes capacitive– and/or inductive relaxations. The charge transfer processes associate with capacitor, inductor, and resistor in the equivalent circuit. For this reason, the plot is successfully used to distinguish the electrolyte response from the electrode polarization as well as ion transfer from electron transfer. The electrolyte and charge transfer resistances can thus be read directly from the plot. The arc in any frequency range corresponds to a rate of any physical or chemical relaxation process which contributes to an overall reaction[26,81]. For example, the arc in low frequency region corresponds to a slow process. Moreover, the Nyquist plot can present the Warburg impedance in low frequency region as a straight line at

45° corresponding to Equation (S12) when ϕ = -45°[26,29,81,90]. The Bode plot presents a variation of phase angle and Z with frequency which can be used to determine relaxation processes by distinguishing relaxation frequencies[26]. Moreover, the plot can provide information about chemical stability of material in terms of an electronic relaxation time (or electron lifetime or time constant) (τ_r). For example, much τ_r reflects high chemical stability and corrosion resistance. τ_r can be calculated from a relaxation frequency (f_r) corresponding to the maximum–phase angle as given by[35,81]:

$$\tau_r = \frac{1}{2\pi f_r} \qquad \text{(S13)}$$

The Bode plots in Fig. 3b show a transition of rate determining step from the uncharged diffusion process at low carburizing power to the gas conversion and ion migration processes at high carburizing power. For conversion process associated with the surface development due to carburizing, the development promoted O_2 concentration gradient and decreased O_2 diffusion range in the solution, which resulted in the decreasing diffusion impedance and decreasing time constant. According to the diffusion model proposed by Primdahl and Mogensen[34], diffusion (perpendicular to electrode surface) of uncharged active specie due to its concentration gradient in stagnant gas layer can generate diffusion impedance indicated by the arc in frequency range of 10 – 1,000 Hz as shown in Fig. 3a. As active specie, O_2 diffused down the concentration gradient which arisen between the steel surface and a distance l over the steel surface at which the O_2 concentration equal to a bulk solution. The bulk solution outside the stagnant layer possessed higher concentration than at the steel surface. High concentration gradient promoted high diffusion of O_2 which resulted in decreasing diffusion impedance.

References (continued from the main article)

55. Dong, H. S–phase surface engineering of Fe–Cr, Co–Cr and Ni–Cr alloys. *Int. Mater. Rev.* **55,** 65–98 (2010).

56. Boonruang, C. & Thong–on, A. Tribological behavior of Ti–5Al–2.5Sn, Ti–10V–2Fe–3Al and Ti–38Al carburized via current heating technique with graphite powders. *Mater. Trans.* **55,** 1073–1082 (2014).

57. Boonruang, C., Kumpangkeaw, W., Sopunna, K., Chomsaeng, N. & Narksitipan, S. Effect of carburizing via current heating technique on the near–surface microstructure of AISI 1020 steel. *Chiang Mai J. Sci.* **39,** 254–262 (2012).

58. Boonruang, C. & Thongtem, S. Surface modification of TiAl alloy via current heating technique. *Appl. Surf. Sci.* **256,** 484–488 (2009).

59. Park, M. H. *et al*. Ferroelectricity and antiferroelectricity of doped thin HfO_2–based films. *Adv. Mater*. **27,** 1811–1831 (2015).

60. Kurpaska, L. *et al*. Influence of Ar–irradiation on structural and nanomechanical properties of pure zirconium measured by means of GIXRD and nanoindentation techniques. *J. Mol. Struct.* **1126,** 226–231 (2016).

61. Peng, J. *et al*. Residual stress gradient analysis by the GIXRD method on CVD tantalum thin films. *Surf. Coat. Technol.* **200,** 2738–2743 (2006).

62. Joardar, J., Kim, S. W. & Kang, S. GI–XRD studies on surface structure of ultrafine $Ti(C_{0.5}N_{0.5})$–WC–Ni cermets at high temperature. *Wear* **261**, 360–366 (2006).

63. Dudognon, J., Vayer, M., Pineau, A. & Erre, R. Mo and Ag ion implantation in austenitic, ferritic and duplex stainless steels: A comparative study. *Surf. Coat. Technol.* **203,** 180–185 (2008).

64. Feng, K., Wang, Y., Li, Z. & Chu, P. K. Characterization of carbon ion implantation induced graded microstructure and phase transformation in stainless steel. *Mater. Charact*. **106,** 11–19 (2015).

65. Pattanasattayavong, P. *et al*. Hole–transporting transistors and circuits based on the transparent inorganic semiconductor copper (I) thiocyanate (CuSCN) processed from solution at room temperature. *Adv. Mater.* **25,** 1504–1509 (2013).

66. Zhou, Y., Han, S.–T., Xu, Z.–X. & Roy, V. A. L. Controlled ambipolar charge transport through a self–assembled gold nanoparticle monolayer. *Adv. Mater.* **24,** 1247–1251 (2012).

67. Wang, Z.–K. *et al*. High efficiency Pb–In binary metal perovskite solar cells. *Adv. Mater.* **28,** 6695–6703 (2016).

68. Yu, X. *et al*. Ultra–flexible, "invisible" thin–film transistors enabled by amorphous metal oxide/polymer channel layer blends. *Adv. Mater.* **27,** 2390–2399 (2015).

69. Wieczerzak, K. *et al*. The effect of temperature on the evolution of eutectic carbides and $M_7C_3 \rightarrow M_{23}C_6$ carbides reaction in the rapidly solidified Fe–Cr–C alloy. *J. Alloys Compd.* **698,** 673–684 (2017).

70. Wulff, H., Quaas, M. & Steffen, H. Investigation of plasma–deposited ITO films by GIXR and GIXRD. *Thin Solid Films* **355–356,** 395–400 (1999).

71. Tsuchiya, M., Sankaranarayanan, S. K. R. S. & Ramanathan, S. Photon–assisted oxidation and oxide thin film synthesis: A review. *Prog. Mater. Sci.* **54,** 981–1057 (2009).

72. Chiu, M.–Y., Jeng, U–S., Su, C.–H., Liang, K. S. & Wei, K.–H. Simultaneous use of small– and wide–angle X–ray techniques to analyze nanometerscale phase separation in polymer heterojunction solar cells. *Adv. Mater.* **20,** 2573–2578 (2008).

73. Rahaei, M. B., Yazdani rad, R., Kazemzadeh, A. & Ebadzadeh, T. Mechanochemical synthesis of nano TiC powder by mechanical milling of titanium and graphite powders. *Powder Technol.* **217,** 369–376 (2012).

74. Sun, Y. *et al*. Flexible n–type high–performance thermoelectric thin films of

poly(nickel–ethylenetetrathiolate) prepared by an electrochemical method. *Adv. Mater.* **28,** 3351–3358 (2016).

75. Pan, F., Song, C., Liu, X. J., Yang, Y. C. & Zeng, F. Ferromagnetism and possible application in spintronics of transition–metal–doped ZnO films. *Mater. Sci. Eng. R* **62,** 1–35 (2008).

76. Franke, R. *et al.* An X–ray photoelectron and X–ray absorption spectroscopic study of colloidal $[Mn^0 \cdot 0.3THF]_x$. *Adv. Mater.* **10,** 126–132 (1998).

77. Bianconi, A. Xanes Spectroscopy in *X–Ray absorption principles, applications, techniques of EXAFS, SEXAFS and XANES* (eds Koningsberger, D. C. & Prins, R.) 573–662 (Wiley, Newyork, 1988).

78. Gaur, A., Shrivastava, B. D. & Nigam, H. L. X–ray absorption fine structure (XAFS) spectroscopy – A review. *Proc. Indian Natn. Sci. Acad.* **79,** 921–966 (2013).

79. Liu, S., Odette, G. R. & Segre, C. U. Evidence for core–shell nanoclusters in oxygen dispersion strengthened steels measured using X–ray absorption spectroscopy. *J. Nucl. Mater.* **445,** 50–56 (2014).

80. Wang, Y.–J., Fang, B., Li, H., Bi, X. T. & Wang, H. Progress in modified carbon support materials for Pt and Pt–alloy cathode catalysts in polymer electrolyte membrane fuel cells. *Prog. Mater. Sci.* **82,** 445–498 (2016).

81. Cesiulis, H., Tsyntsaru, N., Ramanavicius, A. & Ragoisha, G. The study of thin films by electrochemical impedance spectroscopy in *Nanostructures and thin films for multifunctional applications technology, properties and devices* (eds Tiginyanu I., Topala P. & Ursaki V.) 3–42 (Springer, Switzerland, 2016).

82. Zhou, Y. *et al.* Enabling prominent high–rate and cycle performances in one lithium–sulfur battery: Designing permselective gateways for Li^+ transportation in Holey–CNT/S cathodes. *Adv. Mater.* **27,** 3774–3781 (2015).

83. Haase, M. F., Grigoriev, D. O., Möhwald, H. & Shchukin, D. G. Development of

nanoparticle stabilized polymer nanocontainers with high content of the encapsulated active agent and their application in water–borne anticorrosive coatings. *Adv. Mater.* **24,** 2429–2435 (2012).

84. Lu, S.-Y. *et al*. The effect of tempering temperature on the microstructure and electrochemical properties of a 13 wt.% Cr-type martensitic stainless steel. *Electrochim. Acta* **165,** 45–55 (2015).

85. Hirschorn, B. *et al*. Constant-Phase-Element Behavior Caused by Resistivity Distributions in Films. *J. Electrochem. Soc.* **157 (12),** C458–C463 (2010).

86. Yoo, J.-H., Ahn, S.-H., Kim, J.-G. & Lee S.-Y. Influence of target power density and substrate bias voltage on the electrochemical properties of type 304 SS films prepared by unbalanced magnetron sputtering. *Surf. Coat. Technol.* **157,** 47–54 (2002).

87. Xing, X., Han, Z., Wang, H. & Lu, P. Electrochemical corrosion resistance of CeO_2–Cr/Ti coatings on 304 stainless steel via pack cementation. *J. Rare Earths* **33,** 1122–1128 (2015).

88. Wang, N., Fu, W., Zhang, J., Li, X. & Fang, Q. Corrosion performance of waterborne epoxy coatings containing polyethylenimine treated mesoporous–TiO_2 nanoparticles on mildsteel. *Prog. Org. Coat.* **89,** 114–122 (2015).

89. Marcelin, S., Pébère, N. & Régnier, S. Electrochemical characterisation of a martensitic stainless steel in a neutral chloride solution. *Electrochim. Acta* **87,** 32– 40 (2013).

90. Wu, H. *et al*. Flexible and binder–free organic cathode for high–performance lithium–ion batteries. *Adv. Mater.* **26,** 3338–3343 (2014).
